# Resistance against two lytic phage variants attenuates virulence and antibiotic resistance in *Pseudomonas aeruginosa*

**DOI:** 10.3389/fcimb.2023.1280265

**Published:** 2024-01-17

**Authors:** Juan Carlos García-Cruz, Xareni Rebollar-Juarez, Aldo Limones-Martinez, Cristian Sadalis Santos-Lopez, Shotaro Toya, Toshinari Maeda, Corina Diana Ceapă, Lucia Blasco, María Tomás, Clara Estela Díaz-Velásquez, Felipe Vaca-Paniagua, Miguel Díaz-Guerrero, Daniel Cazares, Adrián Cazares, Melisa Hernández-Durán, Luis Esaú López-Jácome, Rafael Franco-Cendejas, Fohad Mabood Husain, Altaf Khan, Mohammed Arshad, Rosario Morales-Espinosa, Ana María Fernández-Presas, Frederic Cadet, Thomas K. Wood, Rodolfo García-Contreras

**Affiliations:** ^1^ Departamento de Microbiología y Parasitología, Facultad de Medicina, Universidad Nacional Autónoma de México (UNAM), Mexico City, Mexico; ^2^ Universidad Univer Milenium, Toluca de Lerdo, Mexico; ^3^ Department of Biological Functions Engineering, Graduate School of Life Science and Systems Engineering, Kyushu Institute of Technology, Kitakyushu, Japan; ^4^ Microbiology Laboratory, Chemistry Institute, Universidad Nacional Autónoma de México (UNAM), Mexico City, Mexico; ^5^ Microbiología Traslacional y Multidisciplinar (MicroTM), Instituto de Investigación Biomédica (INIBIC), Universidad de A Coruña (UDC), A Coruña, Spain; ^6^ Servicio de Microbiología, Hospital A Coruña (CHUAC), Universidad de A Coruña (UDC), A Coruña, Spain; ^7^ Laboratorio Nacional en Salud, Diagnóstico Molecular y Efecto Ambiental en Enfermedades Crónico-Degenerativas, Facultad de Estudios Superiores (FES) Iztacala, Universidad Nacional Autónoma de México, Tlalnepantla de Baz, Estado de México, Mexico; ^8^ Subdirección de Investigación Básica, Instituto Nacional de Cancerología, Ciudad de México, Mexico; ^9^ Department of Biology, University of Oxford, Oxford, United Kingdom; ^10^ Parasites and Microbes Programme, Wellcome Sanger Institute, Hinxton, United Kingdom; ^11^ Laboratorio de Microbiología Clínica, División de Infectología, Instituto Nacional de Rehabilitación, Luis Guillermo Ibarra Ibarra, Mexico, Mexico; ^12^ Departamento de Biología, Facultad de Química, Universidad Nacional Autónoma de México (UNAM), Mexico City, Mexico; ^13^ Subdirección de Investigación Biomédica, Instituto Nacional de Rehabilitación Luis Guillermo Ibarra Ibarra, Mexico, Mexico; ^14^ Department of Food Science and Nutrition, King Saud University, Riyadh, Saudi Arabia; ^15^ Department of Pharmacology, College of Pharmacy, King Saud University, Riyadh, Saudi Arabia; ^16^ Dental Health Department, College of Applied Medical Sciences, King Saud University, Riyadh, Saudi Arabia; ^17^ PEACCEL, Artificial Intelligence Department, AI for Biologics, Paris, France; ^18^ Department of Chemical Engineering, Pennsylvania State University, University Park, PA, United States

**Keywords:** virulence, tradeoffs, biofilm, phage resistance, phage therapy

## Abstract

**Background:**

Bacteriophage therapy is becoming part of mainstream Western medicine since antibiotics of clinical use tend to fail. It involves applying lytic bacteriophages that self-replicate and induce cell lysis, thus killing their hosts. Nevertheless, bacterial killing promotes the selection of resistant clones which sometimes may exhibit a decrease in bacterial virulence or antibiotic resistance.

**Methods:**

In this work, we studied the *Pseudomonas aeruginosa* lytic phage φDCL-PA6 and its variant φDCL-PA6α. Additionally, we characterized and evaluated the production of virulence factors and the virulence in a Galleria mellonella model of resistant mutants against each phage for PA14 and two clinical strains.

**Results:**

Phage φDCL-PA6α differs from the original by only two amino acids: one in the baseplate wedge subunit and another in the tail fiber protein. According to genomic data and cross-resistance experiments, these changes may promote the change of the phage receptor from the O-antigen to the core lipopolysaccharide. Interestingly, the host range of the two phages differs as determined against the *Pseudomonas aeruginosa* reference strains PA14 and PAO1 and against nine multidrug-resistant isolates from ventilator associated pneumonia.

**Conclusions:**

We show as well that phage resistance impacts virulence factor production. Specifically, phage resistance led to decreased biofilm formation, swarming, and type III secretion; therefore, the virulence towards Galleria mellonella was dramatically attenuated. Furthermore, antibiotic resistance decreased for one clinical strain. Our study highlights important potential advantages of phage therapy’s evolutionary impact that may be exploited to generate robust therapy schemes.

## Introduction

Bacteriophages are the most abundant biological entities, outnumbering bacteria by about an order of magnitude; hence, their influence on bacterial ecology and physiology is enormous, shaping microbiomes. It is estimated that phages kill around 40% of all existing bacteria each day in some places like the ocean and are responsible for a massive amount of genetic exchange interchange among bacteria in diverse environments, from freshwater to gut microbiomes (Keen, 2015). Also, the use of phages as therapeutic agents for combating bacterial recalcitrant infections is growing due to the increased prevalence of multi- and pan-drug resistant pathogenic bacteria that are untreatable with current antimicrobials.

Although phage therapy has many advantages, one of the possible drawbacks of the therapeutic utilization of bacteriophages is that bacteria readily become resistant to them. In principle, phage resistance can be achieved by multiple mechanisms such as abortive infections, avoiding the entrance of phage genetic material or by specialized systems that protect against phage infections, such as the restriction enzyme/methylation systems and CRISPR-Cas (Gordillo Altamirano and Barr, 2019) as well as toxin/antitoxin systems. However, among the resistance mechanisms, the most common is the selection of mutations that decrease or preclude the expression of phage receptors, or otherwise those that change them so that the phage affinity of phages to the host decreases. For *Pseudomonas aeruginosa*, the main phage receptors are the type IV pili and the LPS (Markwitz et al., 2021).

To overcome bacterial resistance toward phages, therapies use a mixture of several different phages, thus decreasing the probability that mutations generating simultaneous resistance against all the phages present in the cocktail arise (Vaitekenas et al., 2021). Although the emergence of phage resistance to phages is considered a major limitation of phage therapy, the acquisition of phage resistance has been associated with a lower expression of a receptor or mutated receptors, which often has a fitness cost for the bacterium (McGee et al., 2023). For example, if phage resistance is through the loss of function of phage receptors, then bacteria could lose important properties such as twitching motility if the receptor is type IV pili or swimming and swarming motility if the receptor is the flagellum (Gurney et al., 2020; Esteves and Scharf, 2022). Phage resistance may also decrease stress tolerance and resistance against detergents, some antibiotics and immune response if the receptor is LPS or capsular components. Moreover, losing capsular components may increase bacterial susceptibility to phagocytosis (Mangalea and Duerkop, 2020; Gordillo Altamirano et al., 2021; Zulk et al., 2022). Furthermore, a severe decrease in antibiotic resistance can be a tradeoff derived from bacteriophage resistance if the receptor is part of a component that actively provides antibiotic resistance, such as the porins of the Resistance-Nodulation-Division efflux pump systems (Chan et al., 2016; Luong et al., 2020).

A notable attribute of bacteriophages with small genomes is that they replicate very fast, leading to an accelerated evolutionary process (Hatfull and Hendrix, 2011; Borin et al., 2021). Therefore, in this work, we used the lytic phage φDCL-PA6 and its variant φDCL-PA6α, that differs only in two amino acids to test if those mutations altered phage infection parameters such as the length of the phages lytic cycle, burst size and host range.

Moreover, we sought to assess whether the development of resistance to phages might result in tradeoffs, leading to a reduction in virulence or antibiotic resistance.

## Material and methods

### Strains and bacteriophages

The *P. aeruginosa* PAO1 and PA14 type strains were obtained from the collection of Dr. Thomas K. Wood from the Pennsylvania State University, and the nine clinical antibiotic-resistant strains (**Supplementary Table S1**) were obtained from the collection of Dr. Rosario Morales Espinosa (and hence named RME) from the Faculty of Medicine UNAM and were isolated from ventilator-associated pneumonia patients. **Supplementary Table S2** and **Supplementary Figure 1** includes detailed information on all the original strains used in this work. φDCL-PA6 phage was isolated from a contaminated river in Temixco, Morelos, Mexico, at coordinates 18°51’13”N 99°13’20”W. The phage variant φDCL-PA6α was obtained from a clear lytic plaque of φDCL-PA6 after sequencing phages obtained from 6 lytic plaques formed in a PA14 strain lawn, five of which gave identical wild-type sequencing results, while one preparation featured four mutations in its genome; and that phage variant was named φDCL-PA6α.

Temperate phage JBD30, donated by Dr. Alan Davison from the University of Toronto was used during the host range screening because it is a well-characterized phage that uses T4P as RBP (Bondy-Denomy et al., 2012).

### Isolation of phage resistant clones

The resistant clones were isolated following the methodology of Gordillo Altamirano and coworkers with few modifications (Gordillo Altamirano et al., 2021). A bacterial lawn was made by mixing 100 µL of bacteria cultures (at turbidity 0.7-1.0) with 4 mL of LB agar (0.7% w/v) previously melted and maintained at a temperature of 45°C. After homogenizing by vortexing, the content was poured into an LB agar plate and allowed to solidify. Once solidified, 25 μL of the corresponding pure phage lysate (phage titer>10^8^ PFU/mL) was dropped and evaporated. After that, the plate was incubated at 37°C overnight. The next day, isolated bacterial colonies grown within the lysis zone were selected and streaked on an LB agar plate. The plate was incubated at 37°C overnight. From this plate, a new isolated bacterial colony was selected and streaked on LB agar. The plate was incubated at 37°C overnight. From this last striatum, cells were harvested in LB liquid medium, mixed with 15% sterile glycerol, and then stored at -70°C. Confirmation of phage resistance was done by generating lawns of the purified clones and spotting the phage to be tested and by making growth curves in the presence of the phages for some strains. A schematic of the procedure and the corresponding growth curves can be found in **Supplementary Figure 2**.

### Genome sequencing

Phages, the parental PA14 strain, and their phage-resistant clones: 14R1-φDCL-PA6, 14R2-φDCL-PA6, 14R1-φDCL-PA6α and 14R2-φDCL-PA6α were sequenced in the Illumina platform as described in (Tostado-Islas et al., 2021). Genomic DNA was extracted from overnight cultures made in LB using the DNeasy UltraClean Microbial Kit (Qiagen, Hilden, Germany). The Nextera XT DNA Sample Prep Kit with TruSeq HT adapters (Illumina, San Diego, CA, USA) was used for the barcoding of the library, and sequencing of the libraries was done with a MiSeq Illumina instrument. The complete genome data was deposited in the GenBank database. The accession number of phage φDCL-PA6 is OR436899. For RME-58, RME-75, and RME-125 the accession numbers are NZ_JAVCYI000000000, NZ_JAVCYJ000000000, and JAWXXI000000000 respectively, and the complete genome sequences of the PA14 resistant clones were included in the GenBank BioProject PRJNA1004870.

### Genome analysis

Sequencing data were processed using the Trimmomatic v.0.39 program (Bolger et al., 2014). Illumina adaptors were eliminated from the FASTQ files with this program, as well as bases below a Phred score of Q30 from the 5’- and 3’ ends. Then the FASTQ files were evaluated with MultiQC.

The phage genome was assembled with the SPAdes v3.13.0 software (Bankevich et al., 2012) and annotated by sequence homology using the DNA Master software (Pope and Jacobs-Sera, 2018) (E-value< 0.001 and using the Standard code) complemented by the manual annotation with HMMER (Potter et al., 2018) and HHpred (Gabler et al., 2020), which predict functions through protein structure. For the HMMER annotation, the default settings were used for the identification of domains (E-value< 0.001 and the BLOSUM62 substitution matrix). For the Hhpred analysis, the default settings were used as well (E-value< 0.001; coverage>20%) and hits greater than 95% of probability were selected for the annotation. The genes annotated are available on **Supplementary Figure 4**.

The family and genus of the phages were determined by sequence homology comparisons with the phage sequences available in the NCBI database. The phage genome graphical representation was constructed by the SnapGene software v.6.2.2 (www.snapgene.com), and the BLAST tool was used to identify the mutated sites in phage φDCL-PA6α (Altschul et al., 1990) by performing the multiple sequence alignment of the phage DNA sequences using the default parameters and with the MSA Viewer the ORFs containing the mutations were identified.

Bacterial genomes were assembled, annotated, and analyzed using the BV-BRC system (Olson et al., 2023). The “Variation Analysis” method was used for identification and annotation of the sequence variations of the resistant clones vs. the PA14 wild-type using the default settings (the aligner was BWM-mem and the SNP caller FreeBayes). The mutations identified with a high impact were selected as relevant.

A phylogenetic tree was generated using the BV-BRC tool (Olson et al., 2023). For the analysis, 100 PATRIC’s global Protein Families (PGFams) were used, and it was performed according to (Fiedler et al., 2021).

### Electron microscopy

The phage particles were centrifuged at 11000 x g for their precipitation for 40 min at 4° C and resuspended in SM buffer (0.1 M NaCl, 1 mM MgSO_4_, 0.2 M Tris-HCl, pH 7.5) (Zabarovsky and Turina, 1988). The phage suspensions were negatively stained with 1% aqueous uranyl acetate before being analyzed by transmission electron microscopy (TEM) in a JEOL JEM-1011 electron microscope.

### Phage adsorption

The adsorption of phages to the bacterial surface receptors of PA14 was determined in triplicate using a multiplicity of infection (MOI) of 0.01, according to the procedures described in (Pacios et al., 2022).

### One-step growth curve assay

A One-step growth curve of phages at a MOI of 0.01, was done in triplicate for the PA14 strain to determine the latent period and the burst size of the phage, following the methodology described in (Bleriot et al., 2022).

### Phages killing curve assays

Killing curves for the phages were performed in LB by following the bacterial turbidity using a Perkin Elmer Victor Nivo plate reader. When the strains reached an early logarithmic phase (turbidity OD_600nm_ 0.3 to 0.4), the cultures were infected with phages at an MOI of 1. The turbidity, the CFU/mL, and the PFU/mL were determined every 30 min for 3 h. In all cases, the control was the strain without phage infection. All analyses were performed in triplicate.

### Phage host range determination

Initially, all phages were amplified in one of the reference strains, PAO1 or PA14, by generating phage stocks with at least 10^8^ PFU/mL.

The host range was determined by the standard spot assay as previously described (Kutter (2009)). using nine clinical antibiotic-resistant strains of *P. aeruginosa*. Saline solution was used as negative control. All determinations were made in triplicate. Strains were considered sensitive when clear spots were present, otherwise they were considered resistant.

### Antibiotic susceptibility

Susceptibility to antibiotics was evaluated by the microdilution method using relevant antibiotics for *P. aeruginosa* (amikacin, ceftazidime, ciprofloxacin, colistin, gentamicin, levofloxacin, meropenem, and piperacillin/tazobactam) at concentrations from 0.062 to 64 μg/mL for all except for piperacillin/tazobactam (128/4–0.125/4 μg/mL). The experimental procedures and antibiotics breakpoints were performed according to the CLSI recommendations (CLSI, 2020).

### Pyocyanin quantification

Pyocyanin was extracted from the supernatants of overnight cultures in an LB medium with chloroform and 0.1 N HCl (Essar et al., 1990). Then, pyocyanin concentrations were determined spectrophotometrically using Beer’s Law by multiplying the absorbance at OD_520nm_ by the molar absorption coefficient of 17.072 (Montelongo-Martínez et al., 2023).

### Exoproteases quantification

Extracellular protease was quantified using aliquots obtained from the supernatants of LB overnight cultures by determining the hydrolysis of azocasein following the procedures described in (Loarca et al., 2019).

### Biofilm production

Overnight cultures of *P. aeruginosa* were diluted with LB medium to a turbidity of OD_600nm_ 0.2 in a 48-well PVC microplate. The plate was then incubated at 37°C without shaking for 24 h. to facilitate biofilm formation. After this incubation period, the bacterial growth was measured by recording the turbidity. The supernatant from the plate was discarded, and the plate was subsequently washed twice with distilled water. Once the plate was dry, it was incubated with methanol for 20 min to fix the biofilms. Subsequently, the plate was stained with a 1% crystal violet solution for 40 min. Following the staining, the plate was washed twice with distilled water, and absolute ethanol was added to dissolve the crystal violet retained by the biofilms. Finally, absorbance at OD_570nm_ was measured, and the results were reported as the absorbance of the biofilm/growth turbidity.

### Swarming motility

Swarming motility assays were performed as previously described (García-Contreras et al., 2020) on 0.5% agar M8 6-well plates (0.2% glucose, 0.5% casamino acids, and 1 mM MgSO_4_). After the agar plates were solidified and dried, 2.5 μL of a cell suspension in distilled water with a turbidity of 0.08 OD_600nm_ was inoculated at each well’s center. The plate was incubated under static conditions at 37°C for 24 h. Subsequently, the plaque was photographed, and the images were analyzed as reported before (Morales-Soto et al., 2015) to quantify the swarming surface.

### T3S protein secretion assay and immunoblotting

*P. aeruginosa* strains were grown overnight in LB broth at 37°C with shaking at 200 rpm. Precultures were used to inoculate (initial turbidity OD_600nm_ 0.05) LB supplemented with 10 mM MgCl_2_, 0.5 mM CaCl_2_ and 5 mM EGTA pH 7.4. Bacterial cultures were grown under the same conditions until they reached a turbidity of OD_600nm_ 0.8. One mL of each culture was centrifuged at 16,300 x g for 2 min. The supernatant was transferred to a 1.5 mL microtube and centrifuged again to remove residual bacteria. Nine-hundred milliliters of supernatant was taken, and 100 mL of trichloroacetic acid (TCA) was added and incubated for 12 h at 4°C. Precipitated proteins from the supernatant were harvested by centrifugation at 18,100 x g at 4°C for 30 min. The resulting protein pellet was resuspended in 1X SDS-Page sample buffer with 10% v/v saturated TRIS, and the buffer sample volume was normalized according to turbidity of each culture. The secreted proteins were solved on 15% SDS-PAGE and transferred to a nitrocellulose membrane, blocked overnight with 5% w/v non-fat milk in TBS-T (Tris buffered saline with 0.1% v/v Tween 20). The membrane was rinsed with TBS-T and probed against anti-ExoU polyclonal antibodies. The immunoblotting was carried-out using a Western Chemiluminescent HRP Substrate Kit (Millipore), and protein bands were visualized on a C-Digit Blot Scanner (Li-Cor).

### *Galleria mellonella* infection

Strains were inoculated in LB medium, cultured overnight at 37°C at 200 rpm, re-inoculated to a turbidity of OD_600nm_ 0.05, and grown until they reached a turbidity of OD_600nm_ 0.5. Cultures were then diluted with sterile saline solution to a turbidity of 0.2 and used to prepare 10-fold serial dilutions: 20 µL of bacterial suspensions containing approximately 20 CFU were injected into each larva, and the larvae were incubated at 37° C without food. Survival was recorded daily for 5 days.

### Statistical analysis

The GraphPad Prism software v. 8.0.1 was used for the graph design and the statistical analysis.

The non-parametric Kruskal-Wallis and *post hoc* Dunn tests for independent groups were used to assess statistical differences in the production of the evaluated virulence factors, with *p*-values < 0.05 considered significant compared to the wild-type group.

The Kaplan–Meier survival curves of *G. mellonella* were analyzed with the Log Rank Mantel–Cox test, and *p*-values < 0.05 were regarded as significant compared to the WT group.

## Results

### Slight changes in the genome of the phage variant alter attachment, latency, and burst size

**Figure 1:**
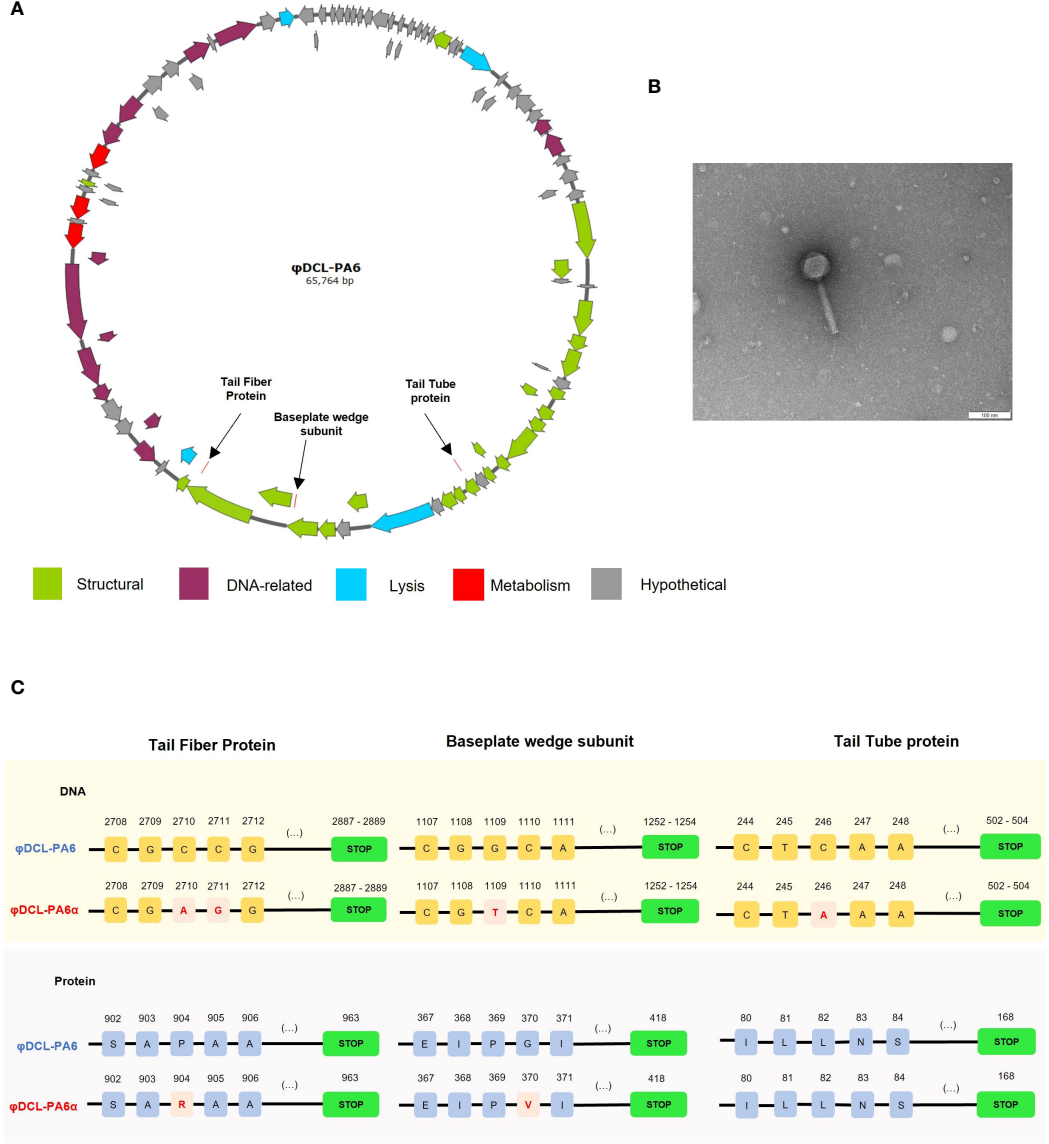
**(A)** Graphical representation of the genome of phage φDCL-PA6 constructed with SnapGene v6.2.2. The black arrows point to the mutations of the phage variant φDCL-PA6α located in three structural genes corresponding to the tail fiber protein, the baseplate wedge protein, and the tail tube protein. **(B)** Transmission electron microscopy (TEM) image of the phage variant φDCL-PA6α that belongs to the order Caudovirales. The TEM scale bar represents 100 nm. **(C)** Mutations identified on the phage variant φDCL-PA6α. Four punctual mutations lead to changes in only two amino acids on the tail fiber protein and the baseplate wedge subunit. Each yellow square represents a nucleotide, while each blue square represents an amino acid, with their respective position on the top. Red squares represent changes in the nucleotide or amino acid sequence. Green boxes represent the stop codon.

Electron microscopy of phage φDCL-PA6 and the spontaneous variant φDCL-PA6α isolated from a φDCL-PA6 plaque of a lawn of *P. aeruginosa* PA14 shows that they have a *Myoviridae* morphology, and the genome analysis showed they belong to the *Caudoviricetes* class, genus *Pbunavirus*, both phages have 64 kb genome length (**Figures 1A, B**). The phages had a GC content of 55.5% and 93 genes (none of which correspond to tRNA genes), and the closest related known phage is *Pseudomonas* phage vB_PaeM_FBPa34 (ON857937.1).

**Figure 2:**
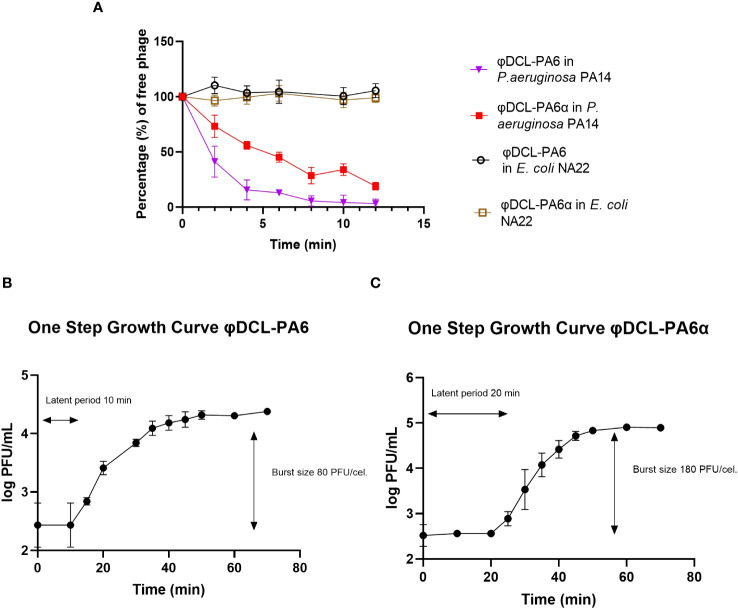
**(A)** Phage adsorption curves of phage φDCL-PA6 and its variant φDCL-PA6α on P. aeruginosa PA14 and E. coli NA22. The adsorption of the phage variant is less effective than the adsorption of the original phage. For future experiments, the adsorption time for both phages was 10 minutes. One-step growth curves for phage φDCL-PA6 **(B)** and its variant φDCL-PA6α **(C)**. The latent period and burst size for phage φDCL-PA6 were 10 minutes and 80 PFU/cell, respectively. For the phage variant φDCL-PA6α, the latent period and burst size were 20 minutes and 180 PFU/cell, respectively. The dots represent the mean of three experiments, and the bars represent the standard deviation.

The genome comparison between them revealed that they differ only in 4 nucleotides. The four mutations led to two amino acid substitutions; one G to T mutation in the gene encoding the baseplate wedge subunit at position 1109 leading to a G to V amino acid substitution at position 370; the other two mutations changed two adjacent C to A and G in the gene encoding the tail fiber protein at positions 2710 and 2711, respectively, leading to a P to R substitution at position 904 of the protein (**Figure 1C**). Phages φDCL-PA6 and φDCL-PA6α were characterized by adsorption and by a one-step infection curve to determine the length of the latency phase and the burst size. Our results revealed that the attachment of the original phage to the PA14 strain was faster and reached a higher percentage than the attachment of the variant φDCL-PA6α (**Figure 2A**), which is consistent with the mutations found in the variant’s tail fiber protein, which may have altered structures important for the phage interaction with its receptor. The latency length of the original phage was shorter than that of the latency for the variant (10 min vs 20 min), but the phage production was 2.25-fold higher for the variant (**Figures 2B, C**). Therefore, we show that two amino acid substitutions in proteins important to the host-phage interaction drastically change the phage´s physiology, promoting a decrease in the attachment ability and increasing the latency period but increasing the number of free viral particles produced per lytic cycle.

### Phage φDCL-PA6 and its variant φDCL-PA6α differ in their host range

The host range of infection of φDCL-PA6, its variant φDCL-PA6α, and the temperate phage JBD30, which uses type IV pili as a receptor (Shah et al., 2021) were determined in the two reference strains as well as in the nine clinical isolates. Remarkably, the phage φDCL-PA6 phage effectively infected the two reference strains and seven of the MDR clinical isolates, while it is unable to infect only the strains RME 124 and RME 125. Interestingly, the phage variant φDCL-PA6α lost the ability to infect the strains RME-22, RME-60, and RME-118 but gained the ability to infect the strain RME-124. In contrast, the temperate JBD30 phage only infected the strains PA14 and RME-124.

### Resistance against the phage φDCL-PA6 and its variant φDCL-PA6α

**Figure 3:**
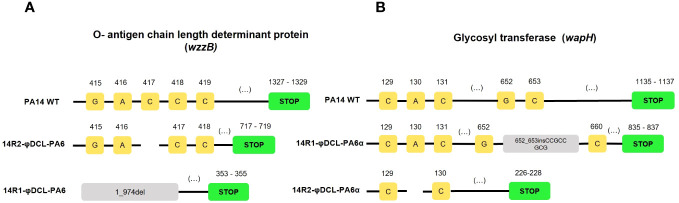
Mutations identified on the P. aeruginosa PA14 resistant clones to phage φDCL-PA6 **(A)** and phage φDCL-PA6α **(B)**. The mutations of phage φDCL-PA6-resistant clones are located in the wzzB gene involved in the O-antigen synthesis, whereas the mutations of phage φDCL-PA6α-resistant clones are located in the wapH gene involved in the LPS core synthesis. Each yellow square represents a nucleotide with its respective position on the top. Blank spaces represent deletions. Green boxes represent the stop codon. Del stands for deletion and ins stands for insertion.

To further characterize the phages φDCL-PA6 and φDCL-PA6α, two resistant *P. aeruginosa* PA14 mutants were obtained against each phage, and cross-resistance was evaluated. We found that the original phage could not infect the two resistant mutants obtained against itself nor those resistant against the φDCL-PA6α variant. In contrast, φDCL-PA6α could not infect the mutants isolated against itself but was able to infect the mutants resistant against the original phage. As a control, the phage JBD30 could infect all resistant mutants (**Supplementary Figure 3**). The genomic analysis of the PA14 mutants resistant to the original phage revealed the presence of a 974 bp deletion of the gene *wzzB*, encoding an O-antigen length determinant protein in one mutant and a base pair deletion at position number 417 of the same gene in the other resistant mutant, shifting the reading frame (**Figure 3A**). The mutants resistant to the phage variant contained a 9 bp insertion at position 652 of the gene *wapH* encoding a glycosyl transferase and a one bp deletion at position 129 in the gene, the same for the other mutant (**Figure 3B**).

### Phage resistance drifts towards attenuated production of virulence factors and reduced virulence in *G. mellonella* for PA14

**Figure 4:**
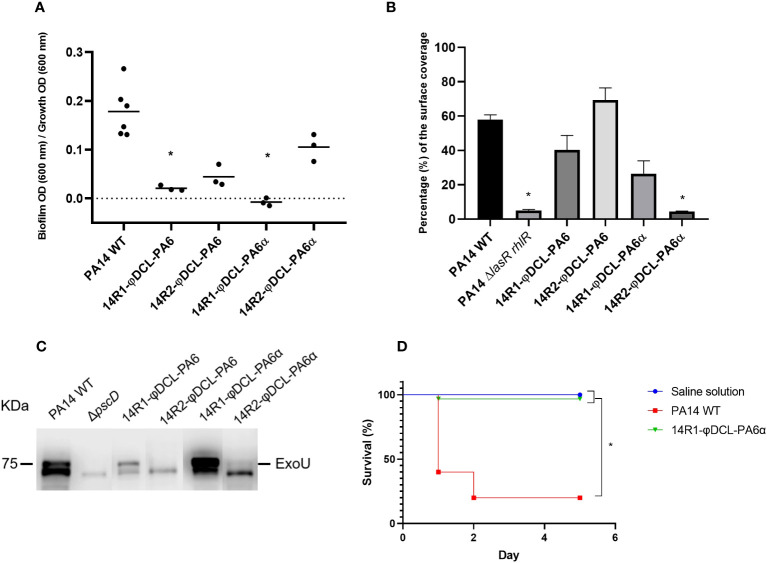
**(A)** Biofilm production of the P. aeruginosa PA14 phage-resistant clones. The dots represent the mean of three experiments. **(B)** Swarming motility assessment of the PA14 WT, the PA14 ΔlasRrhlR mutant, and the phage-resistant clones of the PA14 strain. For the statistical analysis of **(A, B)**, Kruskal-Wallis and Dunn’s tests for independent groups were used (p-values < 0.05 were regarded as significant compared to the PA14 WT group). **(C)** Immunoblotting of the ExoU protein from P. aeruginosa supernatants. The image corresponds to a representative image of three different replicates. **(D)** Kaplan–Meier survival curve of Galleria mellonella infected with P. aeruginosa PA14 WT and the P. aeruginosa PA14 phage-resistant clone 14R1-φDCL-PA6α. Control groups were administered with saline solution. At least 20 larvae were examined per group. Data were analyzed using the Log Rank Mantel–Cox test in GraphPad Prism 8. Significance was determined by the Mantel–Cox test (*, p 0.05).

Since bacterial resistance against phages sometimes results in fitness tradeoffs such as a decrease in virulence factor production, we evaluated the expression of a variety of virulence factors in mutants resistant to the phages φDCL-PA6 or φDCL-PA6α, including biofilm formation, swarming motility, type III secretion, pyocyanin production, and caseinolytic activity. Our results revealed that biofilm formation was severely impaired in all the resistant mutants (**Figure 4A**), whilst swarming motility was diminished significantly only in those resistant to φDCL-PA6α (**Figure 4B**). In addition, the secretion of the ExoU effector by the type III secretion system decreased in the mutants resistant to either phage (**Figure 4V**). In contrast, the resistant mutants’ pyocyanin production and caseinolytic activity were not altered (**Supplementary Figure 5**). Based on the identified impairment in producing key virulence factors by the phage-resistant mutants, we evaluated their virulence against *G. mellonella*. Our findings showed that the survival rate of the larvae injected with the mutant resistant against the phage variant φDCL-PA6α remained at 90% five days post-infection. In contrast, the survival rate of the larvae injected with the PA14 wild-type strain was only 25% (**Figure 4D**). Therefore, our results show that phage resistance leads to substantial tradeoffs affecting bacterial virulence.

### Phage resistance attenuates biofilm formation, antibiotic resistance, and virulence towards *G. mellonella* in multi-drug resistant clinical isolates

**Figure 5:**
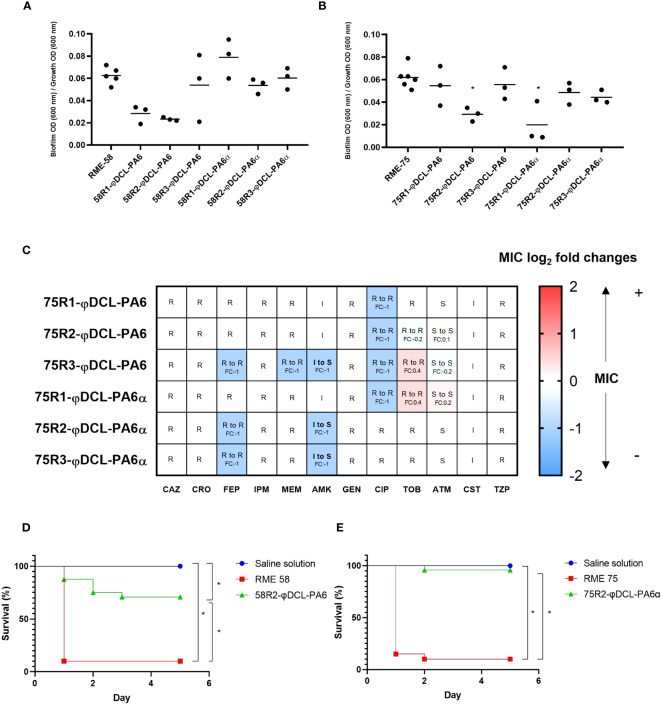
Biofilm production and antibiotic susceptibility profile of XDR *Pseudomonas aeruginosa* phage-resistant clones. Biofilm production of the *P. aeruginosa* RME-58 **(A)** and RME-75 **(B)** phage-resistant clones. The dots represent the mean of three experiments. For the statistical analysis, the Kruskal-Wallis and the Dunn’s tests for independent groups were used (*p*-values < 0.05 were regarded as significant compared to the wild-type groups). **(C)** Heat map of log_2_ fold changes in MIC values against a panel of 14 antibiotics. Heat map intensities for each phage resistant clone represent the fold change in MIC compared to the wild-type RME-75 strain. S, sensitive; I, intermediate; R, resistant; fold change (FC), log_2_ fold changes CAZ: Ceftazidime, CRO: Ceftriaxone, FEP: Cefepime, DOR: Doripenem, IPM: Imipenem, MEM: Meropenem, AMK: Amikacin, GEN: Gentamicin, CIP: Ciprofloxacin, TGC: Tigecycline, TOB: Tobramycin, ATM: Aztreonam, CST: Colistin, TZP: Tazobactam/Piperacilline. Kaplan–Meier survival curves of *G mellonella* infected with *P. aeruginosa* RME-58 **(D)** and RME-75 **(E)** wild type and the *P. aeruginosa* phage-resistant clones, 58R2-φDCL-PA6 and 75R2-φDCL-PA6α, respectively. Control groups were administered with saline solution. At least 20 larvae were examined per group. Data was analyzed using the Log Rank Mantel–Cox test in GraphPad Prism 8. Significance was determined by the Mantel-Cox test (*, *p* 0.05).

We extend our screening to strains representing clinical backgrounds to expand our analysis of the implications and consequences of the evolution towards phage resistance further to the PA14 background. The multi-drug resistance isolates RME-58 and RME-75 both with genomes close to PA14 (**Supplementary Figure 1**) were selected for this analysis, and three resistant mutants (per strain) against both phages. As observed in PA14, we found that the biofilm production of some of the phage resistance isolates showed an impaired formation (**Figures 5A, B**). Furthermore, since the RME strains were extensively drug-resistant (**Supplementary Materials**), we explore a possible phage resistance fitness-cost against their antimicrobial resistance (AMR) levels. Interestingly, the antibiograms showed that the isolate of RME-75 resistant to the φDCL-PA6 phage turned from intermediate resistant to sensitive against amikacin, decreasing its minimum inhibitory concentration (MIC) two-fold. The same clone decreased its resistance against cefepime, meropenem, and ciprofloxacin by two-fold, but was still considered resistant (**Figure 5C**). In contrast, no significant changes were found in the AMR profile for the phage resistance isolates to the strain RME-58 (**Supplementary Figure 6**). Finally, the virulence against *G. mellonella* of the RME-75 phage-resistant mutant to φDCL-PA6 and one of the RME-75 phage resistant mutant to φDCL-PA6α were evaluated. We found that both phage resistance mutants of the different clinical isolates remarkably reduced their virulence towards *G. mellonella* (**Figures 5D, E**). Hence, all these results further support the notion that the phage resistance fitness cost can entail a decreased biofilm formation, attenuated virulence, and reduced antibiotic resistance, which are key features the opportunistic pathogen *P. aeruginosa* use for its infections. This study has important implications for the phage therapy approach, especially regarding the potential positive evolutionary collateral effects drifted by phage treatments targeting bacterial human pathogens.

## Discussion

Bacteriophages are ubiquitous and constantly co-evolving with their bacterial hosts. This work explored the effects of two amino acid changes, one within the baseplate wedge subunit and the other at the tail fiber protein. Such mutations produced a phage variant with an altered host range, diminished absorption, increased latency period length in its replicative cycle, and significantly increased viral particle production (burst size). We hypothesize that the observed changes were due to a change in the phage receptor from the O-antigen to the LPS core. This idea is supported by the mutations found in the resistant strains against the original φDCL-PA6 phage and against the phage variant φDCL-PA6α since the affected gene (*wzzB*) on the resistant clones to phage φDCL-PA6 is involved on the synthesis of the O-antigen while the mutated gene (*wapH*) of the two mutants resistant to the phage φDCL-PA6α is involved on the core synthesis of the LPS.

The gene *wzzB* has more than 99% identity with the gene *wzz2* of *P. aeruginosa* PAO1 and codifies for a polysaccharide co-polymerase protein which determines the length of the LPS by regulating the activity of the O-antigen polymerases (Huszczynski et al., 2019). On the other hand, the gene affected on the clones resistant to the phage φDCL-PA6α has more than 99% identity with the gene *wapH* of *P. aeruginosa* PAO1 and codifies for a glucosyltransferase involved in the addition of a glucose residue on the LPS core (King et al., 2009). In addition to the genomic evidence, the phage cross-resistance experiments showed that the φDCL-PA6α phage was able to infect the φDCL-PA6-resistant clones, while the φDCL-PA6 phage was not able to infect the clones resistant to the φDCL-PA6α phage. Such an observation may be explained by the fact that the O-antigen was not present on the mutated phage-resistant clones because the core of the LPS is necessary for the attachment to that structure (Huszczynski et al., 2020). However, the core of the LPS was assembled on the phage resistant clone to the φDCL-PA6 phage because its synthesis is independent of the O-antigen (Huszczynski et al., 2020).

Interestingly, our results show that biofilm formation, swarming motility, and type III secretion decreased in some phage resistant clones, and we also observed a dramatic reduction of virulence toward *G. mellonella* in the resistant mutants evaluated. Regarding antibiotic resistance, several changes in MICs were observed in the resistant clones, with some showing a decrease and others an increase. However, most of them needed to be more substantial to reclassify the clones into a different category than their parental strains. Nevertheless, we found that one multidrug-resistant strain RME-75, turned from intermediate resistant to sensitive against amikacin and decreased its MIC against the other three antibiotics by 2-fold, suggesting a treatment with these antibiotics would have more chances to succeed for this strain than for the parental one. Our findings agree with previous work that demonstrated how phage resistance in *P. aeruginosa* is often accompanied by fitness compromises such as motility impairment, decrease in biofilm formation ability and resensitization of clinical strains towards some antibiotics (Mangalea and Duerkop, 2020; Markwitz et al., 2021; Castledine et al., 2022; Li et al., 2022; Wannasrichan et al., 2022).

This study demonstrates that mutations in the tail fiber and the baseplate wedge protein of the lytic phage φDCL-PA6 resulted in a shift in its recognition from the O-antigen to the LPS core as the phage receptor. Furthermore, we observed a decrease in the *in vivo* and *in vitro* virulence of certain resistant clones to that phage and its variant, accompanied by resensitization to amikacin in some MDR phage-resistant clones. Taken together, these findings highlight the potential candidacy of the φDCL-PA6 phage for therapeutic use.

Moreover, our discoveries pave the way for exploring phage therapies that go beyond traditional phage cocktails, including the incorporation of phage variants with minor genetic changes yet significant bacterial killing capacities. This extends to alterations in the host range and the use of new receptors.

## Data availability statement

The datasets presented in this study can be found in online repositories. The names of the repository/repositories and accession number(s) can be found below: https://www.ncbi.nlm.nih.gov/genbank/, JAVCYI000000000, https://www.ncbi.nlm.nih.gov/genbank/, JAVCYJ000000000, https://www.ncbi.nlm.nih.gov/genbank/, JAVCYL000000000, https://www.ncbi.nlm.nih.gov/genbank/, JAVCYM000000000, https://www.ncbi.nlm.nih.gov/genbank/, JAVCYN000000000, https://www.ncbi.nlm.nih.gov/genbank/, JAVCYO000000000, https://www.ncbi.nlm.nih.gov/genbank/, OR436899.

## Ethics statement

The manuscript presents research on animals that do not require ethical approval for their study.

## Author contributions

JG-C: Conceptualization, Writing – original draft, Data curation, Investigation, Methodology. XR-J: Investigation, Methodology, Writing – original draft. AL-M: Writing – original draft, Investigation, Methodology. CS: Writing – original draft, Investigation, Methodology. ST: Writing – original draft, Investigation, Methodology. TM: Writing – original draft, Investigation, Methodology, Resources. CC: Writing – original draft, Investigation, Conceptualization, Data curation, Software, Validation. LB: Writing – original draft, Investigation, Methodology. MT: Writing – original draft, Investigation, Methodology, Conceptualization, Resources. CD-V: Writing – original draft, Investigation, Methodology. FV-P: Writing – original draft, Investigation, Methodology, Conceptualization. MD-G: Writing – original draft, Investigation, Methodology. DC: Writing – original draft, Investigation, Methodology, Supervision. AC: Writing – original draft, Investigation, Conceptualization. MH-D: Writing – original draft, Investigation, Methodology. LL-J: Writing – original draft, Investigation, Methodology. RF-C: Writing – original draft, Investigation, Methodology. FH: Writing – original draft, Investigation, Methodology, Resources. AK: Writing – original draft, Investigation, Methodology. MA: Writing – original draft, Investigation, Methodology. RM-E: Conceptualization, Writing – review & editing.AF-P: Methodology, Investigation, Conceptualization, Writing – review & editing. FC: Conceptualization, Resources, Writing – review & editing. TW: Conceptualization, Writing – review & editing, Investigation. RG-C: Conceptualization, Funding acquisition, Project administration, Resources, Supervision, Writing – original draft.

## Supplementary material

Supplementary Table S1 Antibiotic susceptibility profile of the *P. aeruginosa* extensively-drug resistant (XDR) strains RME-58 and RME 75. Minimum inhibitory concentrations (MIC) are expressed in μg/mL.

Supplementary Table S2 Strains used in this work.
