# Erratum: Resistance against two lytic phage variants attenuates virulence and antibiotic resistance in Pseudomonas aeruginosa

**DOI:** 10.3389/fcimb.2024.1391783

**Published:** 2024-03-06

**Authors:** Frontiers Production Office

**Affiliations:** Frontiers Media SA, Lausanne, Switzerland

**Keywords:** virulence, tradeoffs, biofilm, phage resistance, phage therapy

Due to a production error, the captions for **Figures 1**–**4** were mismatched. The corrected Figures and their respective captions appear below.

**Figure 1:**
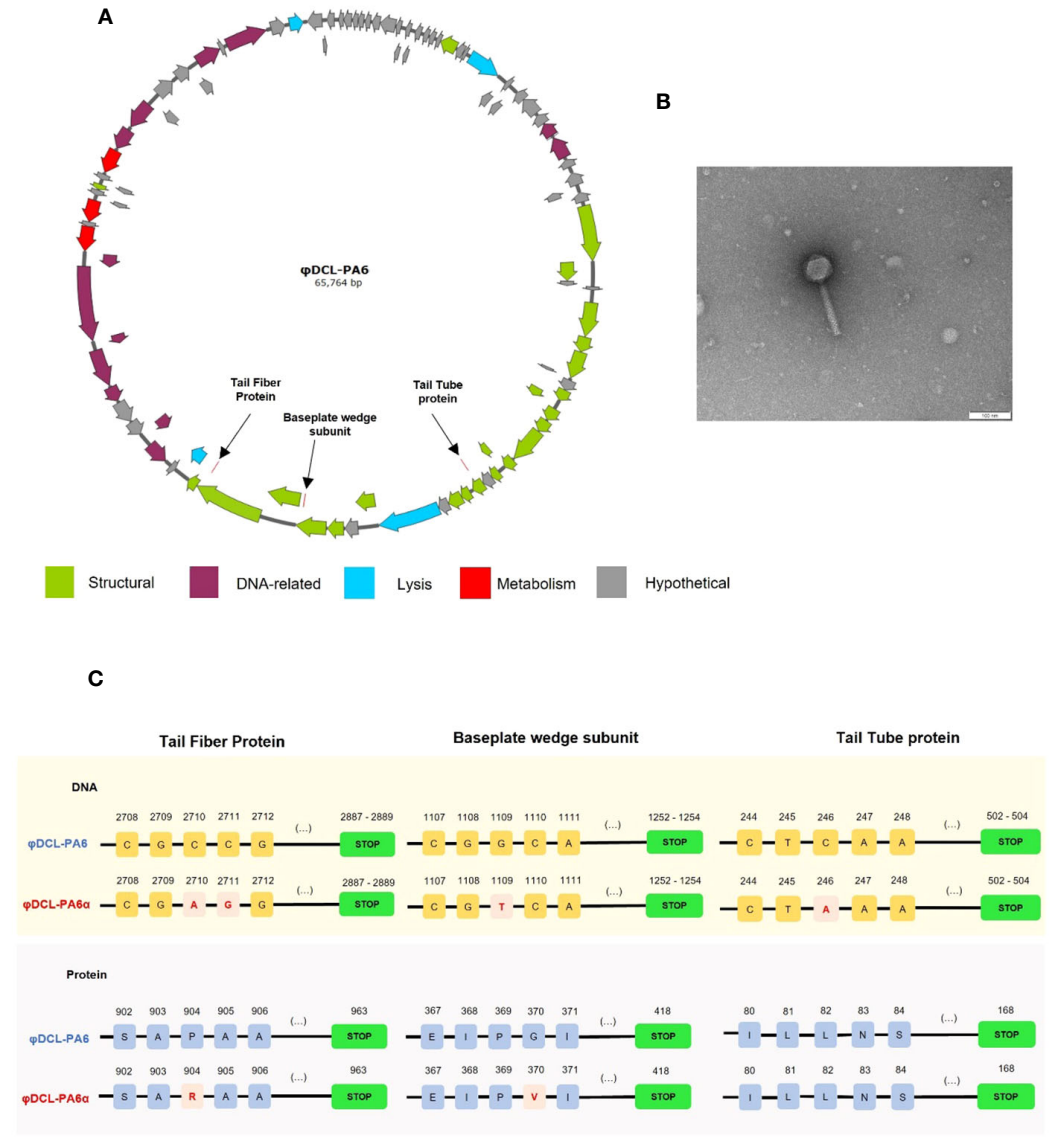
**(A)** Graphical representation of the genome of phage φDCL-PA6 constructed with SnapGene v6.2.2. The black arrows point to the mutations of the phage variant φDCL-PA6α located in three structural genes corresponding to the tail fiber protein, the baseplate wedge protein, and the tail tube protein. **(B)** Transmission electron microscopy (TEM) image of the phage variant φDCL-PA6α that belongs to the order Caudovirales. The TEM scale bar represents 100 nm. **(C)** Mutations identified on the phage variant φDCL-PA6α. Four punctual mutations lead to changes in only two amino acids on the tail fiber protein and the baseplate wedge subunit. Each yellow square represents a nucleotide, while each blue square represents an amino acid, with their respective position on the top. Red squares represent changes in the nucleotide or amino acid sequence. Green boxes represent the stop codon.

**Figure 2:**
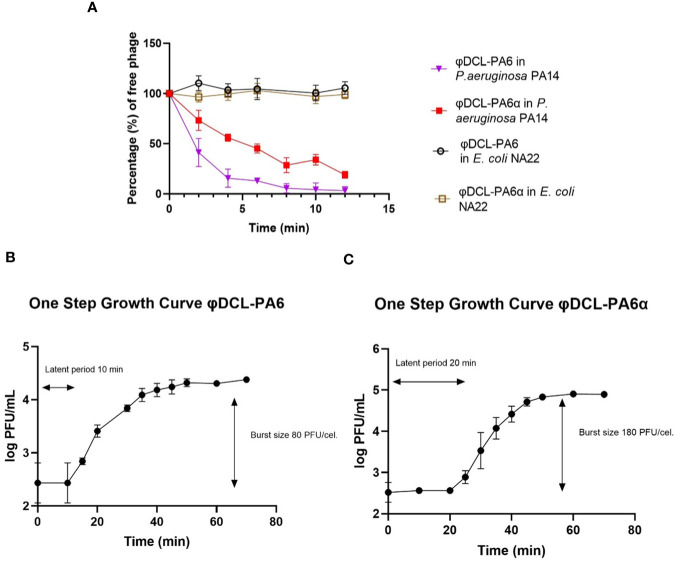
**(A)** Phage adsorption curves of phage φDCL-PA6 and its variant φDCL-PA6α on P. aeruginosa PA14 and E. coli NA22. The adsorption of the phage variant is less effective than the adsorption of the original phage. For future experiments, the adsorption time for both phages was 10 minutes. One-step growth curves for phage φDCL-PA6 **(B)** and its variant φDCL-PA6α **(C)**. The latent period and burst size for phage φDCL-PA6 were 10 minutes and 80 PFU/cell, respectively. For the phage variant φDCL-PA6α, the latent period and burst size were 20 minutes and 180 PFU/cell, respectively. The dots represent the mean of three experiments, and the bars represent the standard deviation.

**Figure 3:**
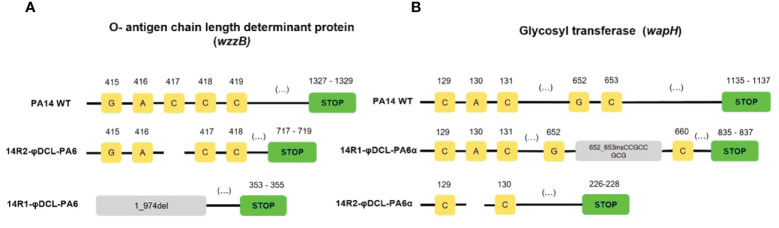
Mutations identified on the P. aeruginosa PA14 resistant clones to phage φDCL-PA6 **(A)** and phage φDCL-PA6α **(B)**. The mutations of phage φDCL-PA6-resistant clones are located in the wzzB gene involved in the O-antigen synthesis, whereas the mutations of phage φDCL-PA6α-resistant clones are located in the wapH gene involved in the LPS core synthesis. Each yellow square represents a nucleotide with its respective position on the top. Blank spaces represent deletions. Green boxes represent the stop codon. Del stands for deletion and ins stands for insertion.

**Figure 4:**
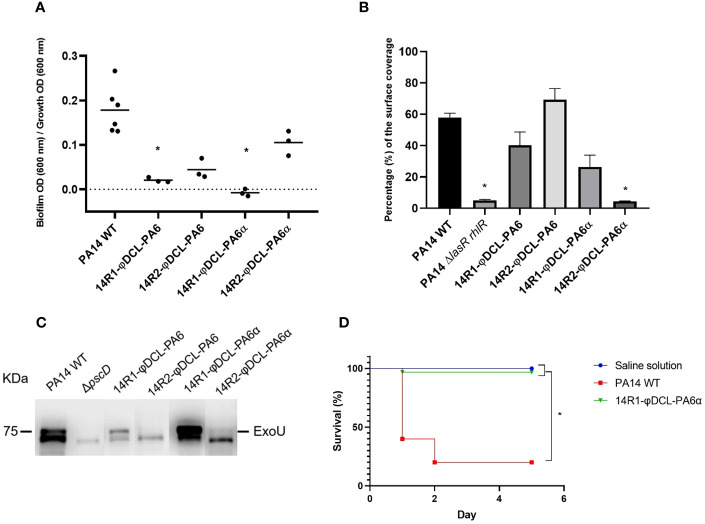
**(A)** Biofilm production of the P. aeruginosa PA14 phage-resistant clones. The dots represent the mean of three experiments. **(B)** Swarming motility assessment of the PA14 WT, the PA14 ΔlasRrhlR mutant, and the phage-resistant clones of the PA14 strain. For the statistical analysis of **(A, B)**, Kruskal-Wallis and Dunn’s tests for independent groups were used (p-values < 0.05 were regarded as significant compared to the PA14 WT group). **(C)** Immunoblotting of the ExoU protein from P. aeruginosa supernatants. The image corresponds to a representative image of three different replicates. **(D)** Kaplan–Meier survival curve of Galleria mellonella infected with P. aeruginosa PA14 WT and the P. aeruginosa PA14 phage-resistant clone 14R1-φDCL-PA6α. Control groups were administered with saline solution. At least 20 larvae were examined per group. Data were analyzed using the Log Rank Mantel–Cox test in GraphPad Prism 8. Significance was determined by the Mantel–Cox test (*, p 0.05).

The publisher apologizes for this mistake.

The original version of this article has been updated.

