# Supplementary material for: Resistance against two lytic phage variants attenuates virulence and antibiotic resistance in Pseudomonas aeruginosa
Source: Front Cell Infect Microbiol. 2024 Jan 17;13:1280265. doi: 10.3389/fcimb.2023.1280265 (PMC10828002; doi:10.3389/fcimb.2023.1280265)
Supplement: Supplementary Table S2 — Strains used in this work. [file Table_2.docx]

| **Supplementary table 2. Strains used in this work** | | | |
| --- | --- | --- | --- |
| **Strain** | **Source** | **Relevant characteristics** | **Accession number** |
| *Pseudomonas aeruginosa* PAO1 | Holloway, B. W. 1955. Genetic recombination in *Pseudomonas aeruginosa*. J. Gen. Microbiol. 13:572-581. | Reference strain | NZ_JAAGAW000000000.1 |
| *Pseudomonas aeruginosa* PA14 | Schroth MN, Cho JJ, Green SK, Kominos SD. Epidemiology of *Pseudomonas aeruginosa* in agricultural areas. In Young VM. (editor) *Pseudomonas aeruginosa: Ecological Aspects and Patient Colonization* New York: Raven Press; 1977 pp. 1–29 | Reference strain | NC_008463.1 |
| *Pseudomonas aeruginosa* RME-22 | Patient with pneumonia | Extensive drug resistance | NA |
| *Pseudomonas aeruginosa* RME-58 |  |  | JAVCYI000000000 |
| *Pseudomonas aeruginosa* RME-60 |  |  | NA |
| *Pseudomonas aeruginosa* RME-66 |  |  | NA |
| *Pseudomonas aeruginosa* RME-75 |  |  | JAVCYJ000000000 |
| *Pseudomonas aeruginosa* RME-101 |  |  | NA |
| *Pseudomonas aeruginosa* RME-118 |  |  | NA |
| *Pseudomonas aeruginosa* RME-124 |  |  | NA |
| *Pseudomonas aeruginosa* RME-125 |  |  | NA |
| *Pseudomonas aeruginosa* PA14 14R1-φDCL-PA6 | This work | PA14 resistant clone 1 to phage φDCL-PA6 | JAVCYL000000000 |
| *Pseudomonas aeruginosa* PA14 Pa14R2-φDCL-PA6 |  | PA14 resistant clone 2 to phage φDCL-PA6 | JAVCYM000000000 |
| *Pseudomonas aeruginosa* PA14 14R1-φDCL-PA6α |  | PA14 resistant clone 1 to the phage variant (φDCL-PA6α) | JAVCYN000000000 |
| *Pseudomonas aeruginosa* PA14 14R2-φDCL-PA6α |  | PA14 resistant clone 2 to the phage variant (φDCL-PA6α) | JAVCYO000000000 |
| *Pseudomonas aeruginosa* RME-58 58R1-φDCL-PA6 | This work | RME-58 resistant clone 1 to phage φDCL-PA6 | NA |
| *Pseudomonas aeruginosa* RME-58 58R2-φDCL-PA6 |  | RME-58 resistant clone 2 to phage φDCL-PA6 | NA |
| *Pseudomonas aeruginosa* RME-58 58R3-φDCL-PA6 |  | RME-58 resistant clone 3 to phage φDCL-PA6 | NA |
| *Pseudomonas aeruginosa* RME-58 58R1-φDCL-PA6α |  | RME-58 resistant clone 1 to the phage variant (φDCL-PA6α) | NA |
| *Pseudomonas aeruginosa* RME-58 58R2-φDCL-PA6α |  | RME-58 resistant clone 2 to the phage variant (φDCL-PA6α) | NA |
| *Pseudomonas aeruginosa* RME-58 58R3-φDCL-PA6α |  | RME-58 resistant clone 3 to the phage variant (φDCL-PA6α) | NA |
| *Pseudomonas aeruginosa* RME-75 75R1-φDCL-PA6 | This work | RME-75 resistant clone 1 to phage φDCL-PA6 | NA |
| *Pseudomonas aeruginosa* RME-75 75R2-φDCL-PA6 |  | RME-75 resistant clone 2 to phage φDCL-PA6 | NA |
| *Pseudomonas aeruginosa* RME-75 75R3-φDCL-PA6 |  | RME-75 resistant clone 3 to phage φDCL-PA6 | NA |
| *Pseudomonas aeruginosa* RME-75 75R1-φDCL-PA6α |  | RME-75 resistant clone 1 to the phage variant (φDCL-PA6α) | NA |
| *Pseudomonas aeruginosa* RME-75 75R2-φDCL-PA6α |  | RME-75 resistant clone 2 to the phage variant (φDCL-PA6α) | NA |
| *Pseudomonas aeruginosa* RME-75 75R3-φDCL-PA6α |  | RME-75 resistant clone 3 to the phage variant (φDCL-PA6α) | NA |
| *Pseudomonas aeruginosa* PA14 *ΔlasR ΔrhlR* | Park, S. Y., Heo, Y. J., Choi, Y. S., Déziel, E., & Cho, Y. H. (2005). Conserved virulence factors of Pseudomonas aeruginosa are required for killing Bacillus subtilis. Journal of microbiology (Seoul, Korea), 43(5), 443–450. | Mutant with disrupted Quorum sensing main regulators | NA |
| *Escherichia coli* O157:H7 strain ZZb1 | Unpublished “Bacteriophage AR1 insensitive E. coli O157:H7 mutants” |  | NZ_JACBNR000000000.1 |
| *Pseudomonas aeruginosa* PA14 Δ*pscD* | Liberati, N. T., Urbach, J. M. *et al*. (2006). An ordered, nonredundant library of Pseudomonas aeruginosa strain PA14 transposon insertion mutants. Proceedings of the National Academy of Sciences of the United States of America, 103(8), 2833–2838. https://doi.org/10.1073/pnas.0511100103 | Mutant strain deficient in the T3SS translocation apparatus | NA |
