# Supplementary material for: Resistance against two lytic phage variants attenuates virulence and antibiotic resistance in Pseudomonas aeruginosa
Source: Front Cell Infect Microbiol. 2024 Jan 17;13:1280265. doi: 10.3389/fcimb.2023.1280265 (PMC10828002; doi:10.3389/fcimb.2023.1280265)
Supplement: Supplementary file 3 [file Presentation_1.pptx]

## Slide 1
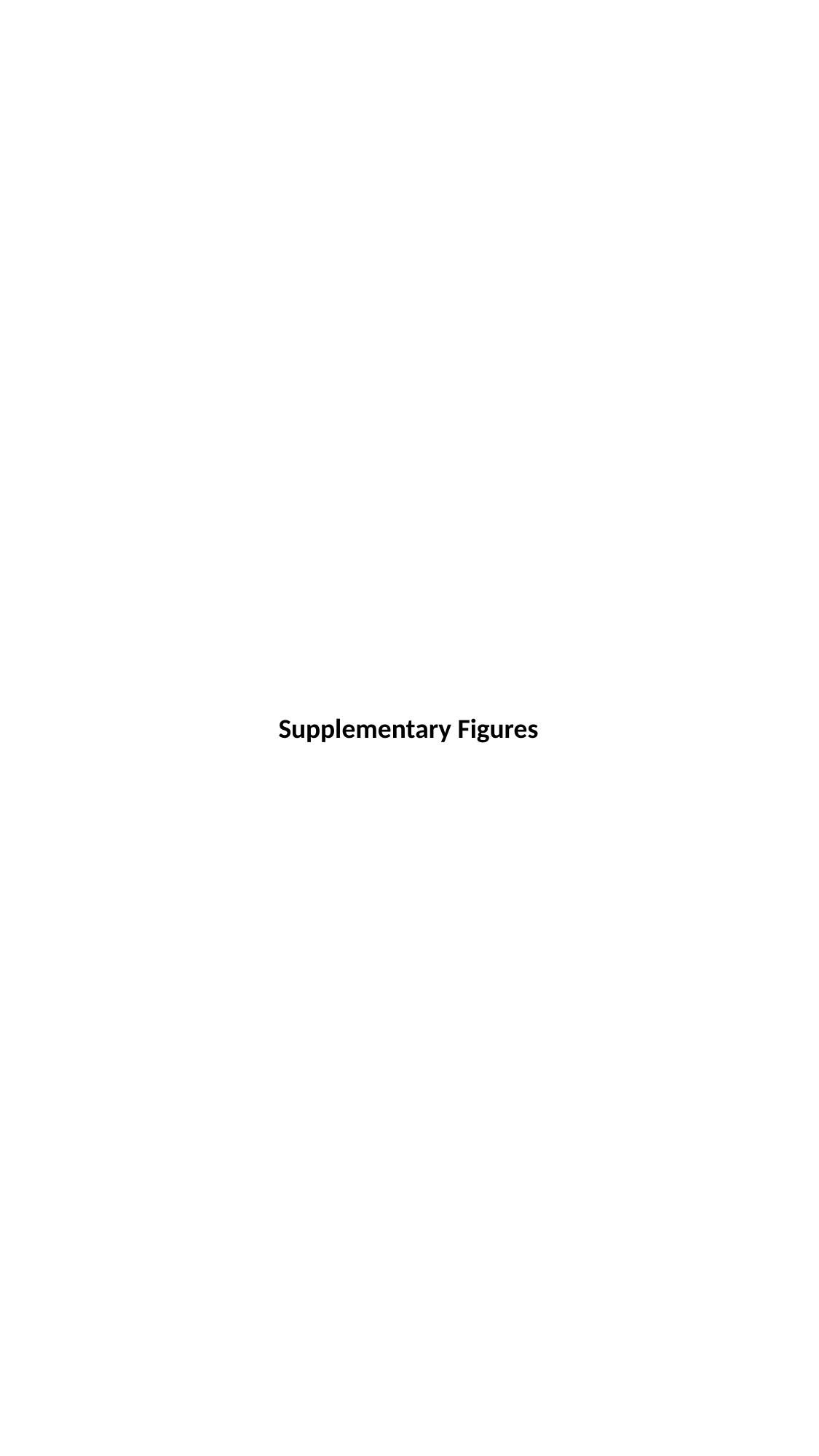

Supplementary Figures

## Slide 2
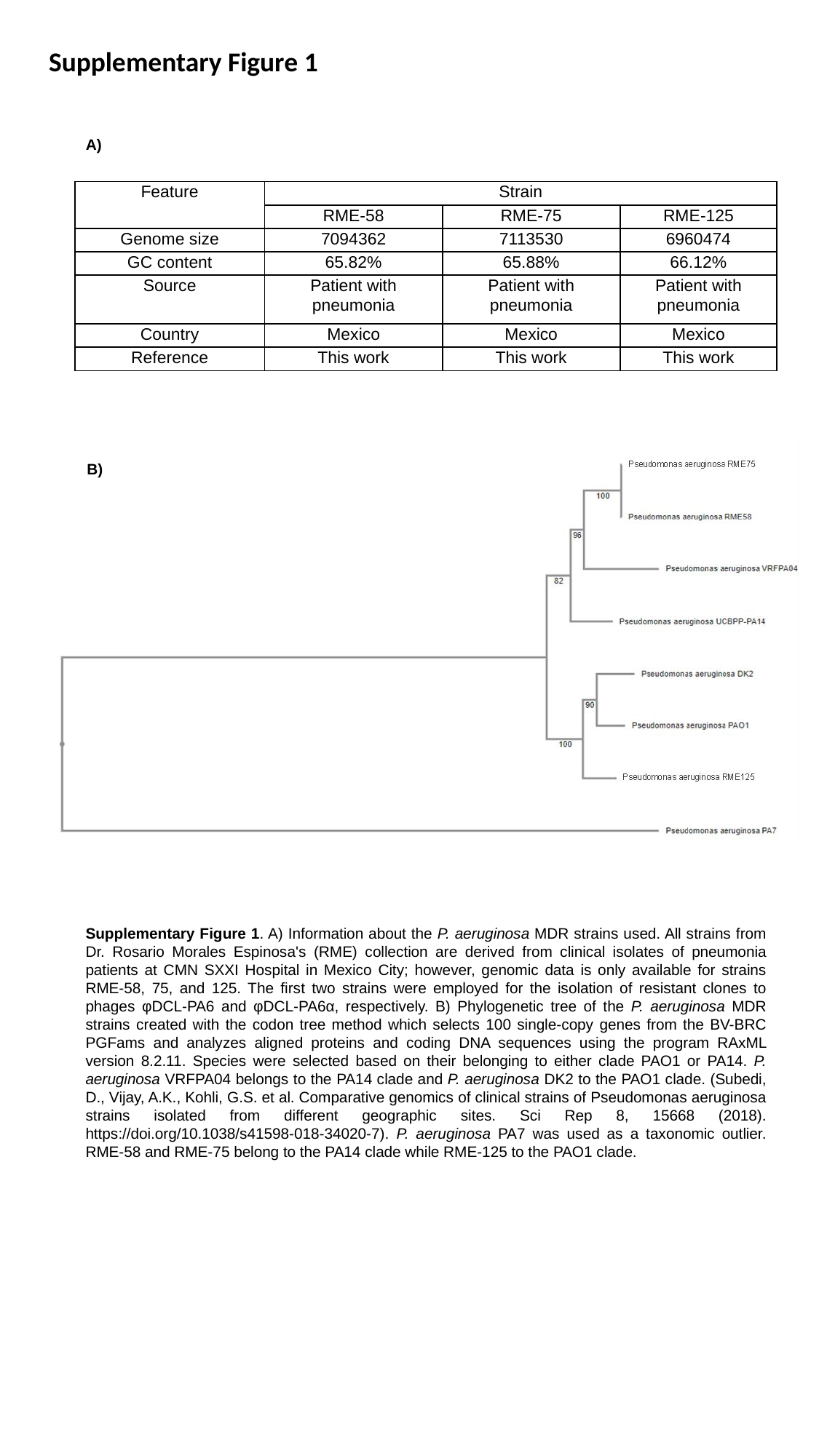

Supplementary Figure 1
A)
| Feature | Strain | | |
| --- | --- | --- | --- |
| | RME-58 | RME-75 | RME-125 |
| Genome size | 7094362 | 7113530 | 6960474 |
| GC content | 65.82% | 65.88% | 66.12% |
| Source | Patient with pneumonia | Patient with pneumonia | Patient with pneumonia |
| Country | Mexico | Mexico | Mexico |
| Reference | This work | This work | This work |
B)
Supplementary Figure 1. A) Information about the P. aeruginosa MDR strains used. All strains from Dr. Rosario Morales Espinosa's (RME) collection are derived from clinical isolates of pneumonia patients at CMN SXXI Hospital in Mexico City; however, genomic data is only available for strains RME-58, 75, and 125. The first two strains were employed for the isolation of resistant clones to phages φDCL-PA6 and φDCL-PA6α, respectively. B) Phylogenetic tree of the P. aeruginosa MDR strains created with the codon tree method which selects 100 single-copy genes from the BV-BRC PGFams and analyzes aligned proteins and coding DNA sequences using the program RAxML version 8.2.11. Species were selected based on their belonging to either clade PAO1 or PA14. P. aeruginosa VRFPA04 belongs to the PA14 clade and P. aeruginosa DK2 to the PAO1 clade. (Subedi, D., Vijay, A.K., Kohli, G.S. et al. Comparative genomics of clinical strains of Pseudomonas aeruginosa strains isolated from different geographic sites. Sci Rep 8, 15668 (2018). https://doi.org/10.1038/s41598-018-34020-7). P. aeruginosa PA7 was used as a taxonomic outlier. RME-58 and RME-75 belong to the PA14 clade while RME-125 to the PAO1 clade.

## Slide 3
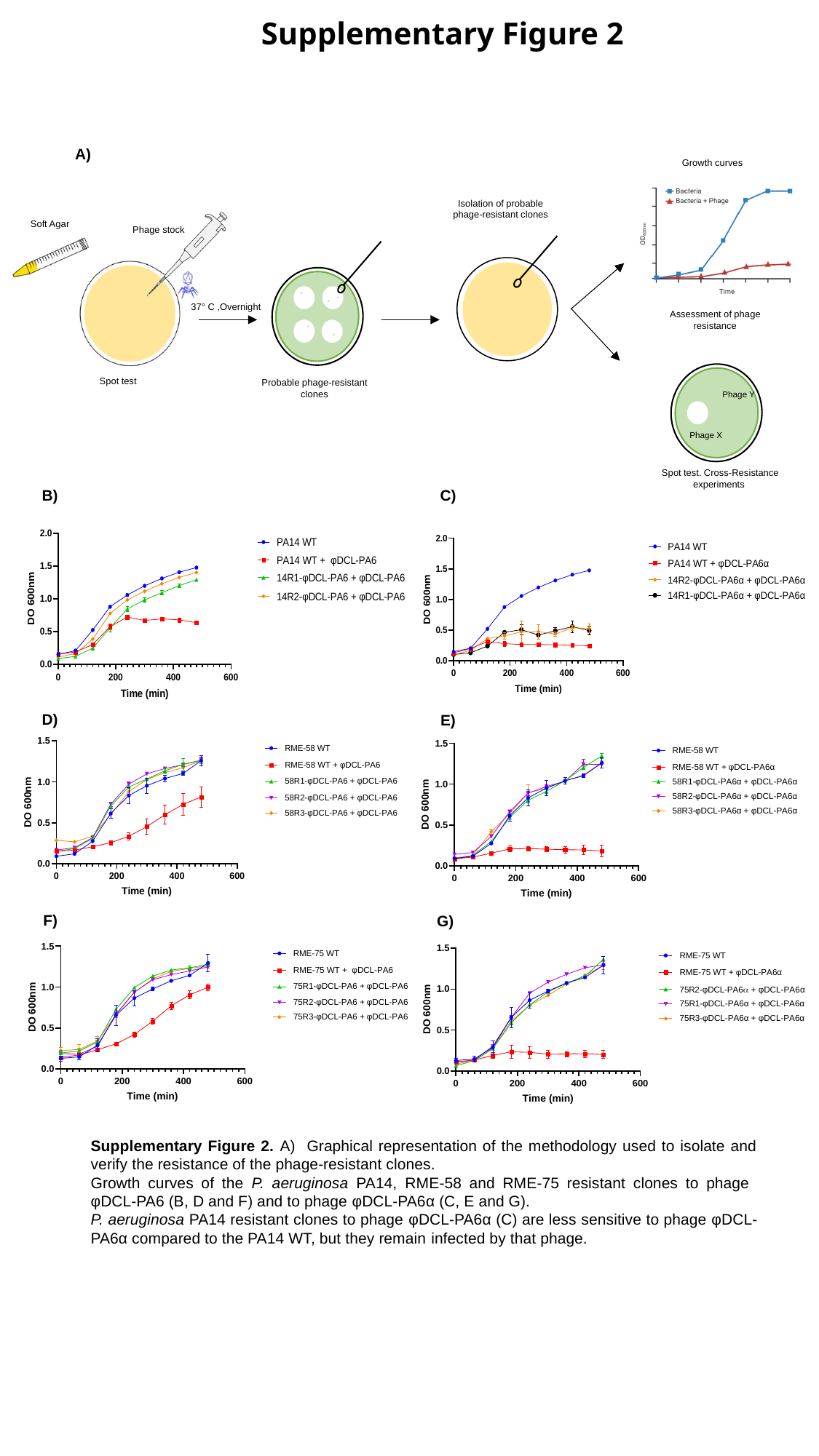

# Supplementary Figure 2
A)
Growth curves
Phage Y
Phage X
Isolation of probable phage-resistant clones
Soft Agar
Phage stock
37° C ,Overnight
Assessment of phage resistance
Spot test
Probable phage-resistant clones
Spot test. Cross-Resistance experiments
C)
B)
D)
E)
F)
G)
Supplementary Figure 2. A) Graphical representation of the methodology used to isolate and verify the resistance of the phage-resistant clones.
Growth curves of the P. aeruginosa PA14, RME-58 and RME-75 resistant clones to phage φDCL-PA6 (B, D and F) and to phage φDCL-PA6α (C, E and G).
P. aeruginosa PA14 resistant clones to phage φDCL-PA6α (C) are less sensitive to phage φDCL-PA6α compared to the PA14 WT, but they remain infected by that phage.

## Slide 4
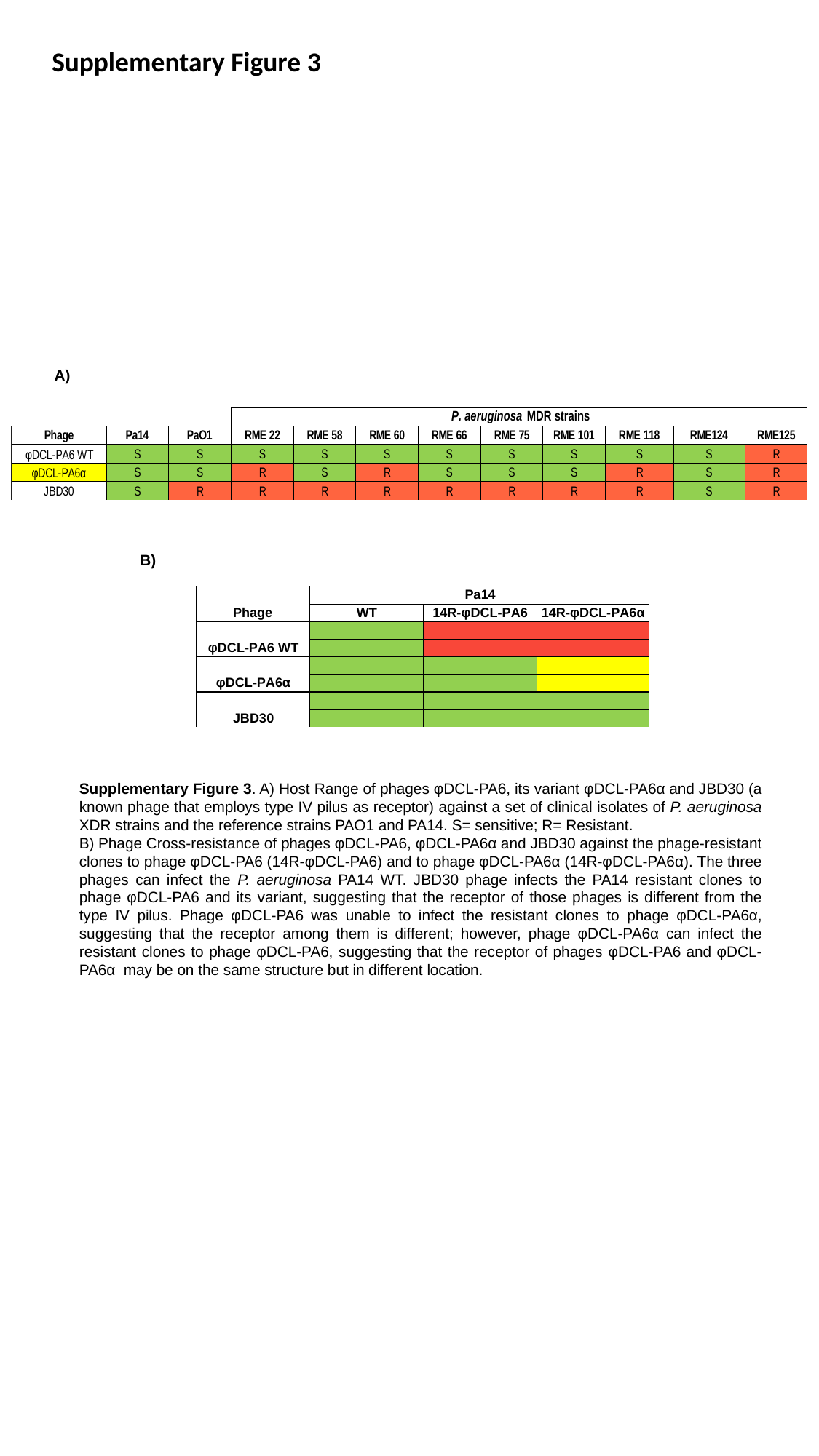

Supplementary Figure 3
A)
B)
Supplementary Figure 3. A) Host Range of phages φDCL-PA6, its variant φDCL-PA6α and JBD30 (a known phage that employs type IV pilus as receptor) against a set of clinical isolates of P. aeruginosa XDR strains and the reference strains PAO1 and PA14. S= sensitive; R= Resistant.
B) Phage Cross-resistance of phages φDCL-PA6, φDCL-PA6α and JBD30 against the phage-resistant clones to phage φDCL-PA6 (14R-φDCL-PA6) and to phage φDCL-PA6α (14R-φDCL-PA6α). The three phages can infect the P. aeruginosa PA14 WT. JBD30 phage infects the PA14 resistant clones to phage φDCL-PA6 and its variant, suggesting that the receptor of those phages is different from the type IV pilus. Phage φDCL-PA6 was unable to infect the resistant clones to phage φDCL-PA6α, suggesting that the receptor among them is different; however, phage φDCL-PA6α can infect the resistant clones to phage φDCL-PA6, suggesting that the receptor of phages φDCL-PA6 and φDCL-PA6α may be on the same structure but in different location.

## Slide 5
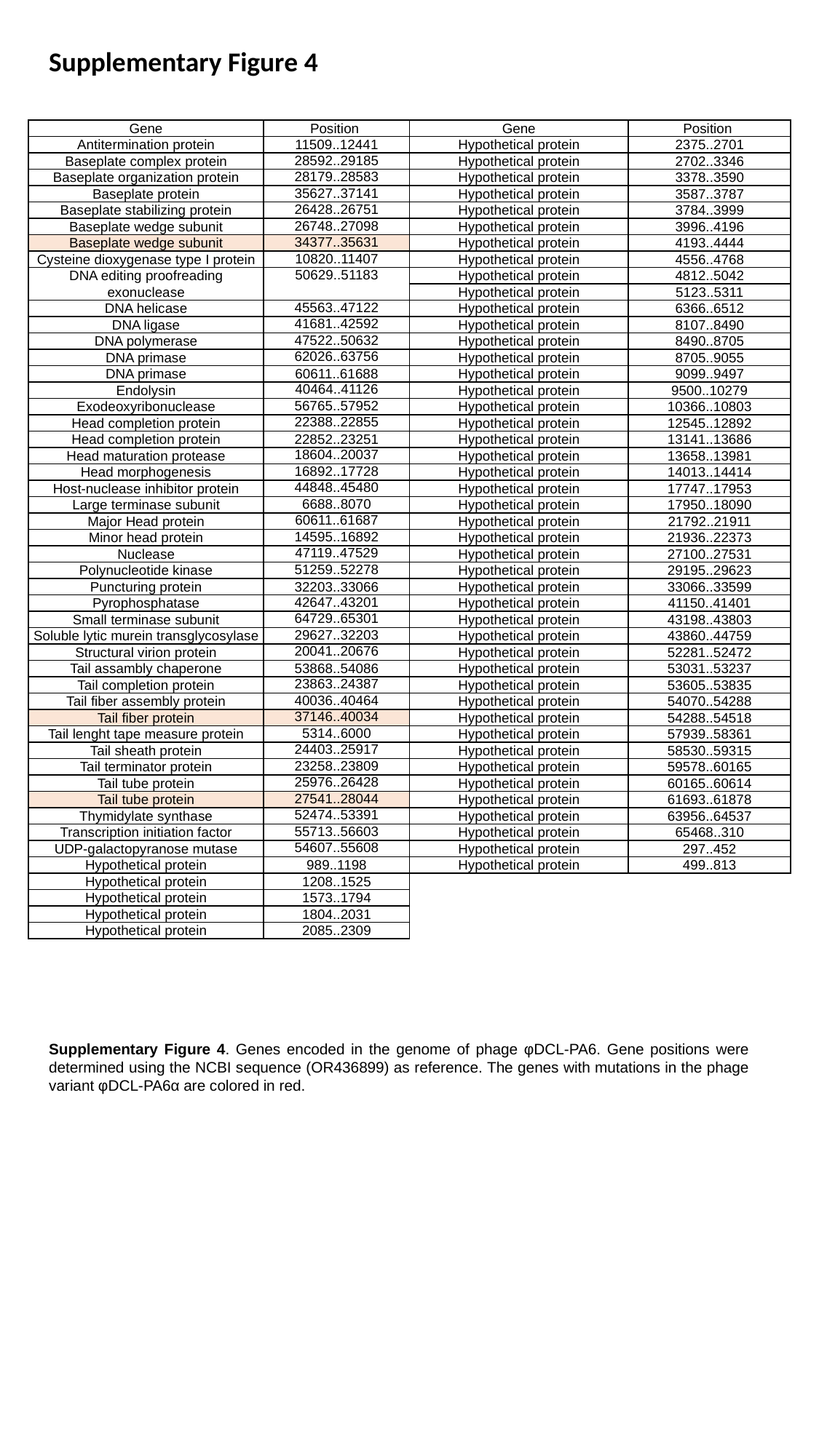

Supplementary Figure 4
| Gene | Position |
| --- | --- |
| Hypothetical protein | 2375..2701 |
| Hypothetical protein | 2702..3346 |
| Hypothetical protein | 3378..3590 |
| Hypothetical protein | 3587..3787 |
| Hypothetical protein | 3784..3999 |
| Hypothetical protein | 3996..4196 |
| Hypothetical protein | 4193..4444 |
| Hypothetical protein | 4556..4768 |
| Hypothetical protein | 4812..5042 |
| Hypothetical protein | 5123..5311 |
| Hypothetical protein | 6366..6512 |
| Hypothetical protein | 8107..8490 |
| Hypothetical protein | 8490..8705 |
| Hypothetical protein | 8705..9055 |
| Hypothetical protein | 9099..9497 |
| Hypothetical protein | 9500..10279 |
| Hypothetical protein | 10366..10803 |
| Hypothetical protein | 12545..12892 |
| Hypothetical protein | 13141..13686 |
| Hypothetical protein | 13658..13981 |
| Hypothetical protein | 14013..14414 |
| Hypothetical protein | 17747..17953 |
| Hypothetical protein | 17950..18090 |
| Hypothetical protein | 21792..21911 |
| Hypothetical protein | 21936..22373 |
| Hypothetical protein | 27100..27531 |
| Hypothetical protein | 29195..29623 |
| Hypothetical protein | 33066..33599 |
| Hypothetical protein | 41150..41401 |
| Hypothetical protein | 43198..43803 |
| Hypothetical protein | 43860..44759 |
| Hypothetical protein | 52281..52472 |
| Hypothetical protein | 53031..53237 |
| Hypothetical protein | 53605..53835 |
| Hypothetical protein | 54070..54288 |
| Hypothetical protein | 54288..54518 |
| Hypothetical protein | 57939..58361 |
| Hypothetical protein | 58530..59315 |
| Hypothetical protein | 59578..60165 |
| Hypothetical protein | 60165..60614 |
| Hypothetical protein | 61693..61878 |
| Hypothetical protein | 63956..64537 |
| Hypothetical protein | 65468..310 |
| Hypothetical protein | 297..452 |
| Hypothetical protein | 499..813 |
| Gene | Position |
| --- | --- |
| Antitermination protein | 11509..12441 |
| Baseplate complex protein | 28592..29185 |
| Baseplate organization protein | 28179..28583 |
| Baseplate protein | 35627..37141 |
| Baseplate stabilizing protein | 26428..26751 |
| Baseplate wedge subunit | 26748..27098 |
| Baseplate wedge subunit | 34377..35631 |
| Cysteine dioxygenase type I protein | 10820..11407 |
| DNA editing proofreading exonuclease | 50629..51183 |
| DNA helicase | 45563..47122 |
| DNA ligase | 41681..42592 |
| DNA polymerase | 47522..50632 |
| DNA primase | 62026..63756 |
| DNA primase | 60611..61688 |
| Endolysin | 40464..41126 |
| Exodeoxyribonuclease | 56765..57952 |
| Head completion protein | 22388..22855 |
| Head completion protein | 22852..23251 |
| Head maturation protease | 18604..20037 |
| Head morphogenesis | 16892..17728 |
| Host-nuclease inhibitor protein | 44848..45480 |
| Large terminase subunit | 6688..8070 |
| Major Head protein | 60611..61687 |
| Minor head protein | 14595..16892 |
| Nuclease | 47119..47529 |
| Polynucleotide kinase | 51259..52278 |
| Puncturing protein | 32203..33066 |
| Pyrophosphatase | 42647..43201 |
| Small terminase subunit | 64729..65301 |
| Soluble lytic murein transglycosylase | 29627..32203 |
| Structural virion protein | 20041..20676 |
| Tail assambly chaperone | 53868..54086 |
| Tail completion protein | 23863..24387 |
| Tail fiber assembly protein | 40036..40464 |
| Tail fiber protein | 37146..40034 |
| Tail lenght tape measure protein | 5314..6000 |
| Tail sheath protein | 24403..25917 |
| Tail terminator protein | 23258..23809 |
| Tail tube protein | 25976..26428 |
| Tail tube protein | 27541..28044 |
| Thymidylate synthase | 52474..53391 |
| Transcription initiation factor | 55713..56603 |
| UDP-galactopyranose mutase | 54607..55608 |
| Hypothetical protein | 989..1198 |
| Hypothetical protein | 1208..1525 |
| Hypothetical protein | 1573..1794 |
| Hypothetical protein | 1804..2031 |
| Hypothetical protein | 2085..2309 |
Supplementary Figure 4. Genes encoded in the genome of phage φDCL-PA6. Gene positions were determined using the NCBI sequence (OR436899) as reference. The genes with mutations in the phage variant φDCL-PA6α are colored in red.

## Slide 6
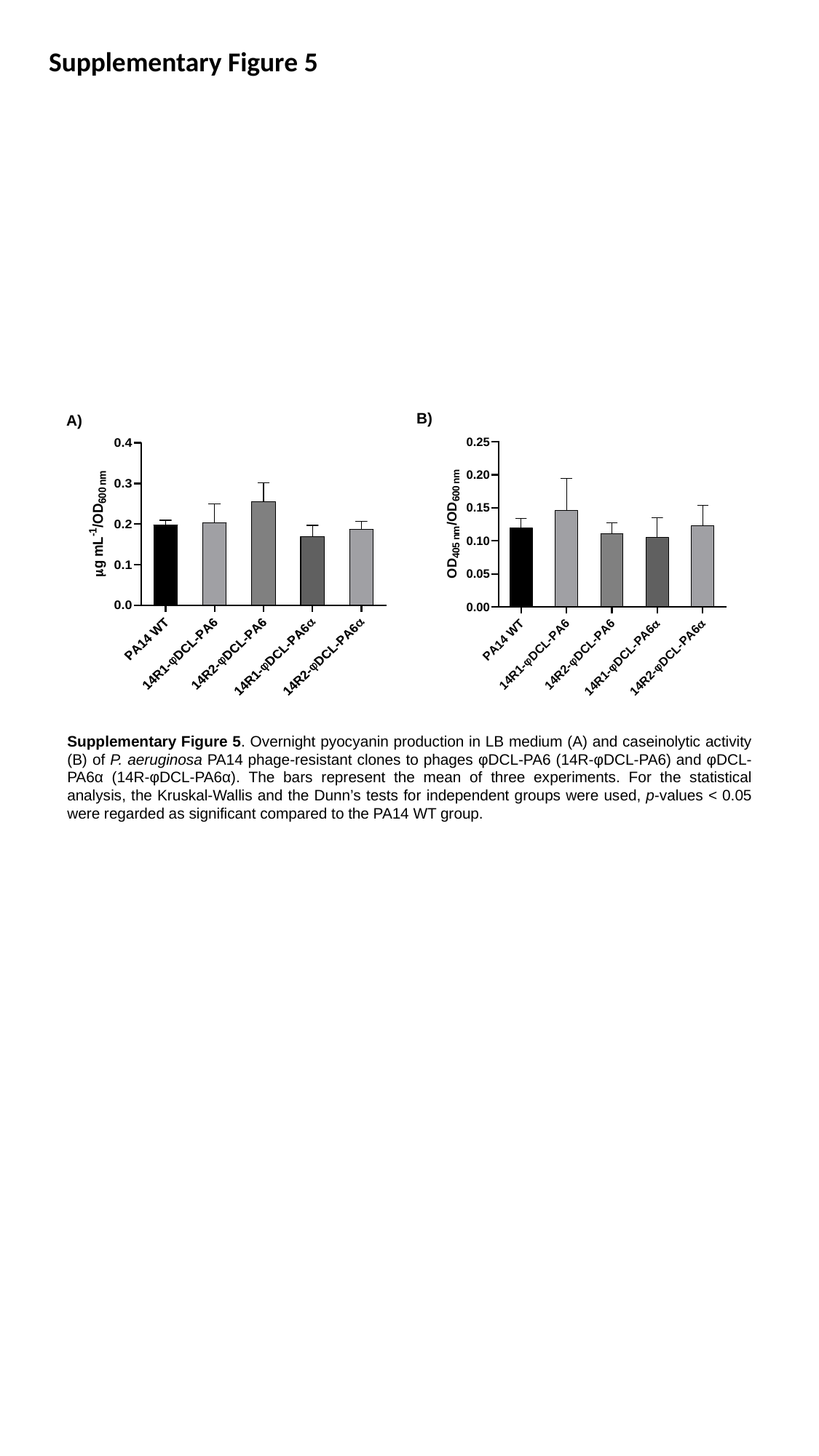

Supplementary Figure 5
B)
A)
Supplementary Figure 5. Overnight pyocyanin production in LB medium (A) and caseinolytic activity (B) of P. aeruginosa PA14 phage-resistant clones to phages φDCL-PA6 (14R-φDCL-PA6) and φDCL-PA6α (14R-φDCL-PA6α). The bars represent the mean of three experiments. For the statistical analysis, the Kruskal-Wallis and the Dunn’s tests for independent groups were used, p-values < 0.05 were regarded as significant compared to the PA14 WT group.

## Slide 7
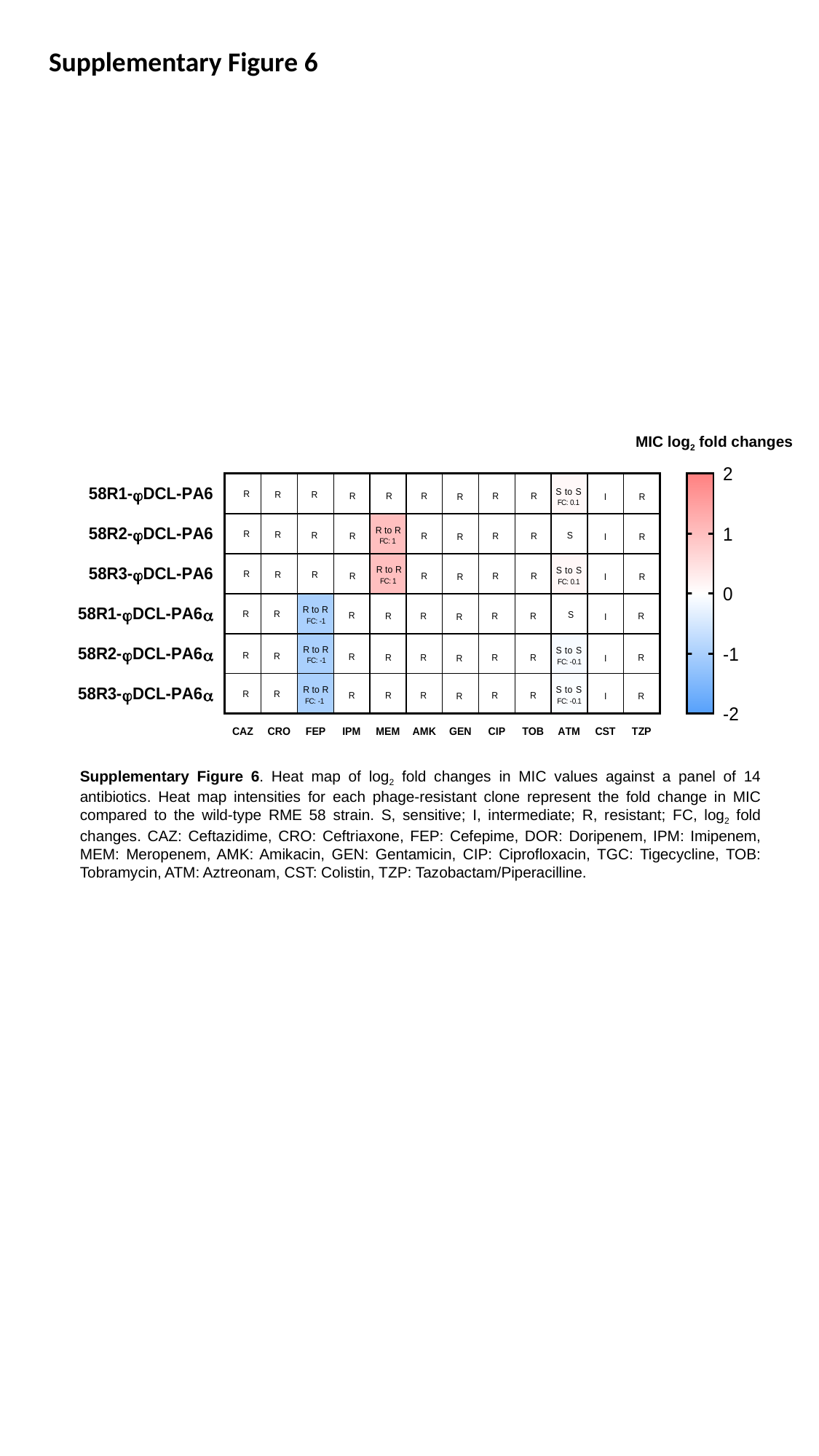

Supplementary Figure 6
MIC log2 fold changes
Supplementary Figure 6. Heat map of log2 fold changes in MIC values against a panel of 14 antibiotics. Heat map intensities for each phage-resistant clone represent the fold change in MIC compared to the wild-type RME 58 strain. S, sensitive; I, intermediate; R, resistant; FC, log2 fold changes. CAZ: Ceftazidime, CRO: Ceftriaxone, FEP: Cefepime, DOR: Doripenem, IPM: Imipenem, MEM: Meropenem, AMK: Amikacin, GEN: Gentamicin, CIP: Ciprofloxacin, TGC: Tigecycline, TOB: Tobramycin, ATM: Aztreonam, CST: Colistin, TZP: Tazobactam/Piperacilline.
